# Hospital Meetings

**Published:** 1905-04-29

**Authors:** 


### HOSPITAL MEETINGS.

### LONDON TEMPERANCE HOSPITAL.

On Wednesday, April 19th, the annual meetings of the London Temperance Hospital were held. The wards were thrown open for inspection in the afternoon, and members of the Board and the matron and sisters conducted visitors over the building.

At 7 P.M. a public meeting was held in one of the empty wards, which had been prettily decorated with palms and yellow flowers. The chair was taken by Alderman J. Vesey Strong, Esq., J.P., Chairman of the Board of Management. He was supported on the platform by Mr. Alderman Purchase, J.P., Mayor of St. Pancras, T. A. Denny, Esq., Professor G. Sims Woodhead, M.D., Sir William Collins (senior surgeon), and others. A large number of supporters and friends were present, and listened with great interest to the Chairman's report of the year's uninterrupted work, and the great need of extensive alterations and additions being at once undertaken in the out-patient department owing to the rapidly increasing numbers of patients. Professor Sims Woodhead followed and traced the change which had taken place in public opinion with regard to the treatment of medical and surgical diseases without the ordinary administration of alcohol.

The total cost of the new buildings which the Board are anxious to erect is estimated at about £15,000, and towards this King Edward's Fund have granted £2,400. Additional funds are urgently needed to meet the increasing work of the hospital in this as in all its departments. The hospital is situated in the midst of a densely populated and extremely poor neighbourhood, and the demands upon its charity continually increase. Last year 1,376 in-patients and 24,721 out-patients were treated.

